# Aminopropylimidazole as an Advantageous Coating in the Synthesis of Functionalized Magnetite Nanoparticles

**DOI:** 10.3390/nano11123276

**Published:** 2021-12-02

**Authors:** Alexandrina Nan, Iolanda-Veronica Ganea, Sergiu Macavei, Rodica Turcu

**Affiliations:** 1 National Institute for Research and Development of Isotopic and Molecular Technologies, 400293 Cluj-Napoca, Romania; iolanda.ganea@itim-cj.ro (I.-V.G.); sergiu.macavei@itim-cj.ro (S.M.); 2 Faculty of Environmental Science and Engineering, “Babeș-Bolyai” University, 400294 Cluj-Napoca, Romania

**Keywords:** magnetite nanoparticles, sodium hydroxide, 1-(3-aminopropyl)imidazole, iron(II) chloride, *X*-ray photoelectron spectroscopy

## Abstract

Implementing new methods to prepare magnetite nanoparticles with a covered or uncovered surface has been, and still is, a significant challenge. In this work, we describe a very clear and effortless way for the preparation of magnetite nanoparticles using two types of bases, namely: 1-(3-aminopropyl)imidazole and sodium hydroxide. Fourier transform infrared spectroscopy (FTIR) served as a tool for the structural investigation of the as-prepared magnetite nanoparticles. The morphology of the samples was investigated using Transmission Electron Microscopy (TEM). Comprehensive high-resolution X-ray photoelectron spectroscopy investigations (XPS) were applied as an effective tool for analyzing the composition of the various types of magnetic nanoparticles. Further polymer linkage was accomplished with poly(benzofuran-co-arylacetic acid) on the amino-functionalized surface of aminopropylimidazole-containing magnetic nanoparticles. The findings are promising for biomedicine, catalysis, and nanotechnology applications.

## 1. Introduction

An intense interest in scientific research and industrial applications is spent developing inorganic nanoparticles due to their unique properties, such as a large surface area/volume ratio and quantum-size effects. Among these, the nanosized iron oxide is a massively promising nanomaterial already used in a wide array of diverse applications, such as catalysis [1,2,3], adsorbent material of pollutants [4,5,6], sensing [7,8], optics [9], drug delivery, and cancer diagnosis and treatment [10,11,12,13]. In this case, the implementation significantly depends on the shape and size of the nanoparticles. For most applications, magnetic nanoparticles (MNP) have to be coated with stabilizers to allow further functionalization and enhance colloidal and chemical stability. Although useful for several applications, hydroxide stabilizers only provide limited opportunities for further functionalization. Amongst other more versatile stabilizers, alkylamines and imidazole were found to be well-known ligands for metal ions. For example, polyvinylimidazole was successfully used to prepare coated magnetite nanoparticles [14]. Magnetite (Fe_3_O_4_) is the most often used magnetic material in the MNP field. It is simply produced from cheap starting materials, usually by coprecipitation, i.e., mixing Fe(III) and Fe(II). As an alternative, mere Fe(II) was used as a starting material for magnetite. In such cases, part of the Fe(II) is oxidized under reaction conditions (oxygen in the air) in an alkaline aqueous solution at low temperatures [15]. For instance, using somewhat harsher conditions, i.e., a higher temperature and longer reaction times, dodecylamine was previously applied in the presence of sodium hydroxide [16,17,18,19]. The technology starting with Fe(II) was not employed for 1-(3-aminopropyl)-imidazole (AIm) as a stabilizer or in combination with sodium hydroxide, neither at an elevated temperature nor at room temperature, and is followed up in the present work.

We synthesized a new MNP starting from Fe(II) using AIm and hydroxide at room temperature in one approach. AIm contains two functional groups: amino and imidazole. It can be assumed that the imidazole moiety of AIm, with its strong, complex properties for metal ions [20,21], is linked to the magnetite surface, while the amino group remained untouched. Thus, it can serve as a linking point for further functionalization. This linking reaction is a straightforward and fast way to obtain functionalized magnetic nanoparticles. Poly(benzofurane-*co*-arylacetic acid) (PBAAA) was used for the attachment of the polymer on the preformed amino-functionalized magnetite, applying a "grafting-from" strategy. Here, the amino groups cause the opening of the lactone rings of PBAAA, which also gives evidence that imidazole is linked to the MNP surface rather than the amino groups of AIm.

## 2. Materials and Methods

### 2.1. Materials

All of the reagents used in this work were acquired from Sigma Aldrich (St. Louis, MO, USA) and Alfa Aesar by ThermoFisher Scientific (Kandel, Germany) and did not need to be further purified. PBAAA (poly(benzofurane-co-arylacetic acid)) was synthesized according to previously published protocols [22].

### 2.2. Preparation of Uncovered Magnetic Nanoparticles (MNP)

A total of 2 M NaOH (60 mL) was added under vigorous stirring at room temperature to a solution formed by dissolving iron(II)chloride tetrahydrate (5.94 g, 30 mmol) in deionized water (150 mL). The mixture was stirred at room temperature for 30 min. After cooling down, the resulting black magnetic solid was magnetically separated and washed repeatedly with distilled water and acetone, yielding 2.68 g magnetite nanoparticles MNP.

### 2.3. Preparation of Magnetic Nanoparticles Stabilized with 1-(3-Aminopropyl)imidazole (MNP-AIm_1)

1-(3-aminopropyl)imidazole AIm (12.5 mL, 100 mmol) was added under vigorous magnetic stirring at room temperature to a solution formed by dissolving iron(II)chloride tetrahydrate (0.8 g, 4 mmol) in deionized water (100 mL). The mixture was allowed under magnetic stirring at room temperature for 30 min. Afterwards, the functionalized magnetic nanoparticles of MNP-AIm_1 were separated magnetically and rinsed with water and acetone. After drying, 0.54 g of MNP-AIm_1 was obtained as black magnetic material.

### 2.4. Preparation of Magnetic Nanoparticles Stabilized with 1-(3-Aminopropyl)imidazole (MNP-AIm_2 in the Presence of Aqueous Sodium Hydroxide)

A total of 2 M NaOH (60 mL) was added to a solution of iron(II)chloride tetrahydrate (5.94 g, 30 mmol) in deionized water (150 mL), followed by the addition of AIm (6.25 mL, 50 mmol) at room temperature. After 30 min, the resulting black magnetic solid was magnetically separated by an external magnet. Next, the solid black material was washed successively with distilled water and acetone, yielding 2.88 g MNP-AIm_2.

### 2.5. Poly(benzofuran-co-arylacetic acid) Linkage on the Amino-Functionalized Magnetic Nanoparticles Surface (MNP-AIm_PAAA)

The magnetic nanoparticles MNP-AIm_1 (0.1 g) were dispersed in distilled water (30 mL), and poly(benzofuran-*co*-arylacetic acid) (0.2 g) was added to the mixture. The reaction mixture was stirred at room temperature for 24 h. The polymer-covered magnetic nanoparticles MNP-AIm_PAAA were separated with an external magnet. After successive washings with methanol and acetone, the magnetic nanostructures of MNP-AIm_PAAA were dried in the oven at 50 °C and further analyzed.

### 2.6. Instrumentation

FTIR (Fourier transform infrared spectroscopy) spectra (400–4000 cm^−1^) were obtained on a pressed tablet prepared from magnetic powder enclosed in KBr, with a JASCO FTIR-6100 spectrophotometer (JASCO Deutschland GmbH, Pfungstadt, Germany). XRD data were collected using a Smart Lab Rigaku diffractometer with a graphite monochromator for Cu-Kα radiation (λ = 1.54 Å) at room temperature. Hitachi H9000NAR (Hitachi Ltd., Tokyo, Japan) and 1010 JEOL transmission electron microscopes (JEOL Ltd., Tokyo, Japan) were used to establish the MNP’s morphology. The chemical surface of the functionalized magnetic nanoparticles was studied using X-ray photoelectron spectroscopy (XPS). An XPS spectrometer (SPECS, Berlin, Germany) containing a dual-anode X-ray source Al/Mg, a PHOIBOS 150 2D CCD hemispherical energy analyzer, and a multi-channeltron detector was employed to record the XPS spectra at a vacuum of 1.9 109 torrs. The XPS spectra were acquired at 30 eV pass energy, with a 0.5 eV/step, using an AlK X-ray source (1486.6 eV) at a power of 200 W. Individual element high-resolution images were obtained by collecting 10–15 scans at 30 eV pass energy with 0.1 eV/step. A Cryogenic Vibrating Sample Magnetometer (Cryogenic Ltd., London, UK) was applied to perform magnetic measurements at room temperature. Thermogravimetry measurements were achieved in the air using TA Instruments SDT Q 600 equipment (TA Instruments Inc., New Castle, DE, USA) at a heating rate of 10 °C min^−1^ within 30 °C to 800 °C.

## 3. Results and Discussion

### 3.1. Synthesis of Magnetic Nanostructure MNP@AIm 

**Scheme 1:**
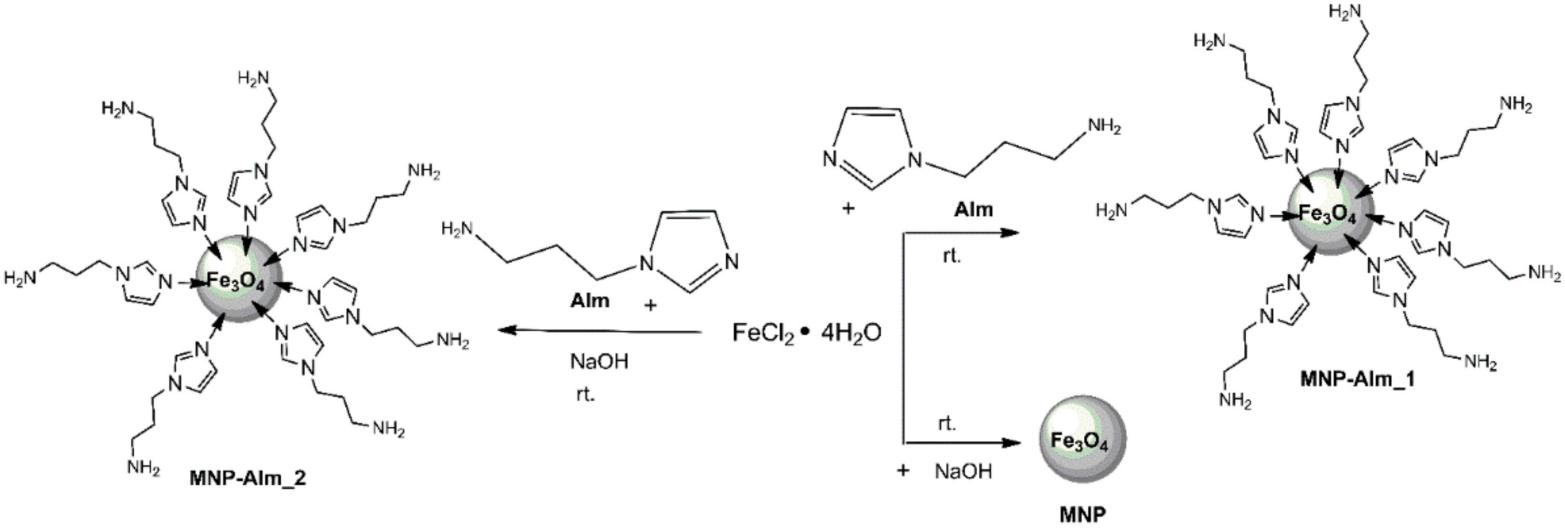
Preparation of magnetite nanoparticles MNP, MNP-AIm_1, and MNP-AIm_2.

We successfully proved that the methodology of using just FeCl_2_ without any Fe(III) source can also be applied in the straightforward synthesis of functionalized magnetite nanoparticles MNP-AIm when AIm, as a stabilizer, is used. Envisaging synthesis at a large scale, it would be advantageous to reduce the quantity of AIm. We first checked that sodium hydroxide could be used as a base in the preparation of magnetic nanoparticles, starting from Fe(II). In fact, MNP was obtained at room temperature after 30 min (Scheme 1). Taking this into consideration, we used the less cost-intensive NaOH as a base and a smaller quantity of AIm and thus obtained MNP-AIm_2 after 30 min at room temperature. All products appeared as magnetic black solids and were quickly separated by an external magnet and purified by washing with water and acetone. They were investigated through the several previously mentioned methods. 

**Scheme 2:**
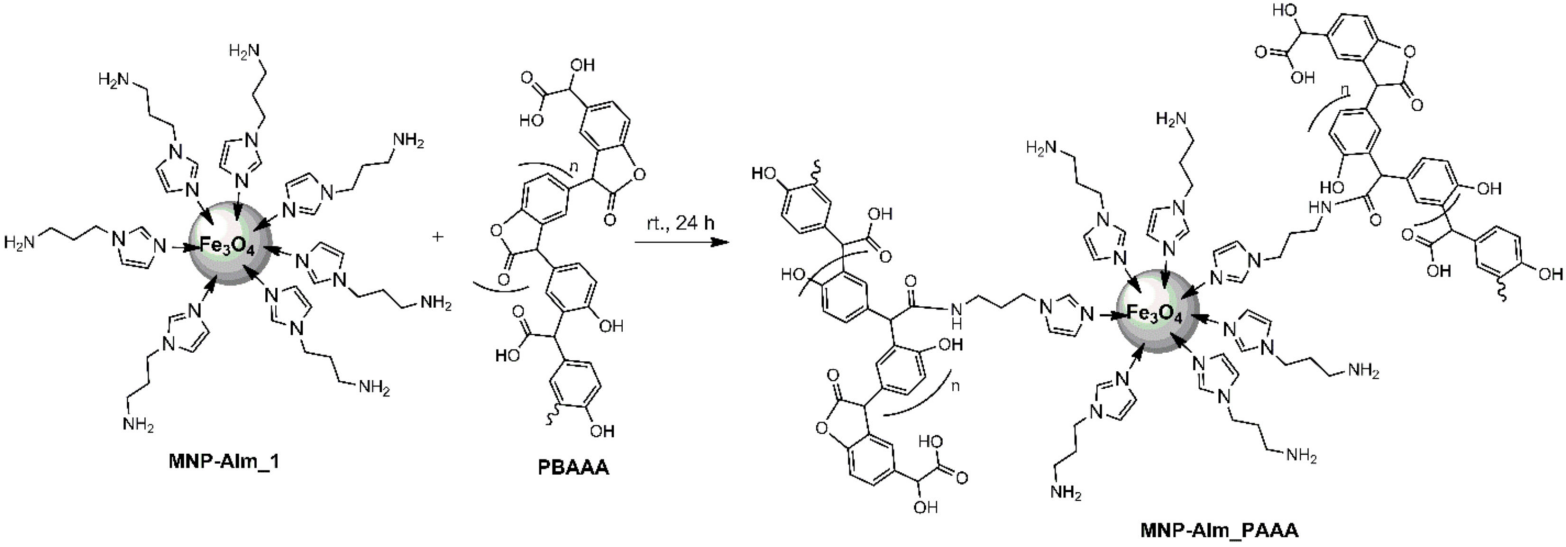
Coating of magnetite nanoparticles MNP-AIm_1 with poly(benzofuran-*co*-arylacetic acid) PBAAA.

Due to the presence of the amino group in AIm molecules on the magnetic nanoparticles surface, one can expect that MNP-AIm_1 serves as a good platform for further immobilization of polymers or other molecules with relevance for practical applications. We demonstrated this potential by linking poly(benzofurane-*co*-arylacetic acid) PBAAA (Scheme 2). This polymer can easily be synthesized in a poly-Friedel-Crafts alkylation by controlled heating of 4-hydroxymandelic acid. It contains lactones, esters, and carboxyl groups. The preparation of magnetic nanoparticles coated with PBAAA consists of two steps: (i) synthesis of amino-functionalized magnetite nanoparticles through a straightforward method; (ii) link of poly(benzofurane-*co*-arylacetic acid) on the surface of these amino-functionalized MNPs, wherein the amino groups open lactone rings, resulting in amide moieties. The amino groups alone caused the opening of the lactone rings in PBAAA, giving evidence that imidazole is linked to the MNP surface rather than the amino groups of AIm.

### 3.2. Characterization of Magnetic Nanostructure MNP@AIm

#### 3.2.1. Infrared Spectroscopy

**Figure 1:**
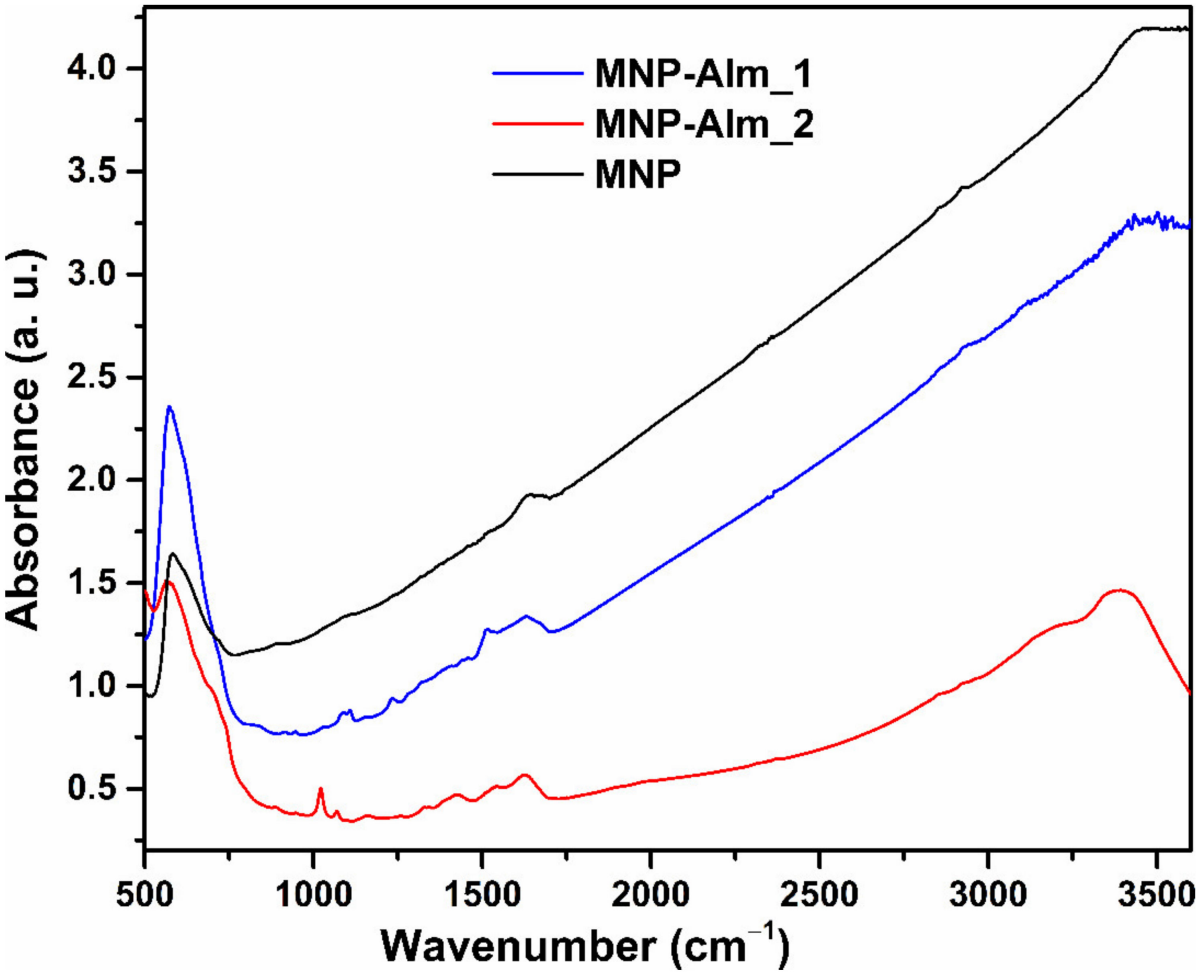
FTIR spectra of uncoated MNP, MNP-AIm_1, and MNP-AIm_2 in KBr pellets.

Figure 1 shows noteworthy changes in the FTIR spectra of coated magnetite MNP-AIm_1 and the MNP-AIm_2 FTIR spectra compared to the uncoated magnetite MNP. In all FTIR spectra, the intensive peak of the specific absorption band of the Fe-O bond is roughly at the same wavelength, namely 580 cm^−1^, and is associated with the stretching vibration of the tetrahedral groups from Fe_3_O_4_. In the MNP-AIm_1 (where only AIm was used for preparation) FTIR spectrum, the absorption bands ascribed to the AIm moiety are slightly more intense than MNP-AIm_2. The -NH_2_ group possesses six normal modes. Not all of them are visible in the present FTIR spectra, the antisymmetric and symmetric stretching modes are at 3586 cm^−1^, and the absorption peak from 1522 cm^−1^ belongs to the in-plane bending vibration of the amine group. The scaled frequency for -NH_2_ rocking mode is assigned at 1107 cm^−1^, and the -NH_2_ wagging mode appears at 890 cm^−1^. In general, the imidazole ring-stretching vibrations have five normal modes. Two frequencies are allocated between 1490 and 1660 cm^−1^, while the other three are between 1300–1000 cm^−1^. At 1660 cm^−1^, the adsorption band that is attributed to the C=N stretching mode can be found. The absorption band from 1440 cm^−1^ ascribes the N-H in-plane bending mode and the methylene scissoring from the imidazole ring. The scaled frequencies, which are associated with the C-H and =C-N bond in-plane bending modes, are found at 1235 cm^−1^. The C-C stretching frequency is located at 1102 cm^−1,^ and the C-N stretching mode is appointed at 950 cm^−1^. The last two mentioned bonds belong to the propylamine chain. The presence of the absorption bands specific to magnetite and those of the AIm component in both FTIR spectra provide evidence of the formation of functional magnetite in MNP-AIm_1, as well as in MNP-AIm_2. 

**Figure 2:**
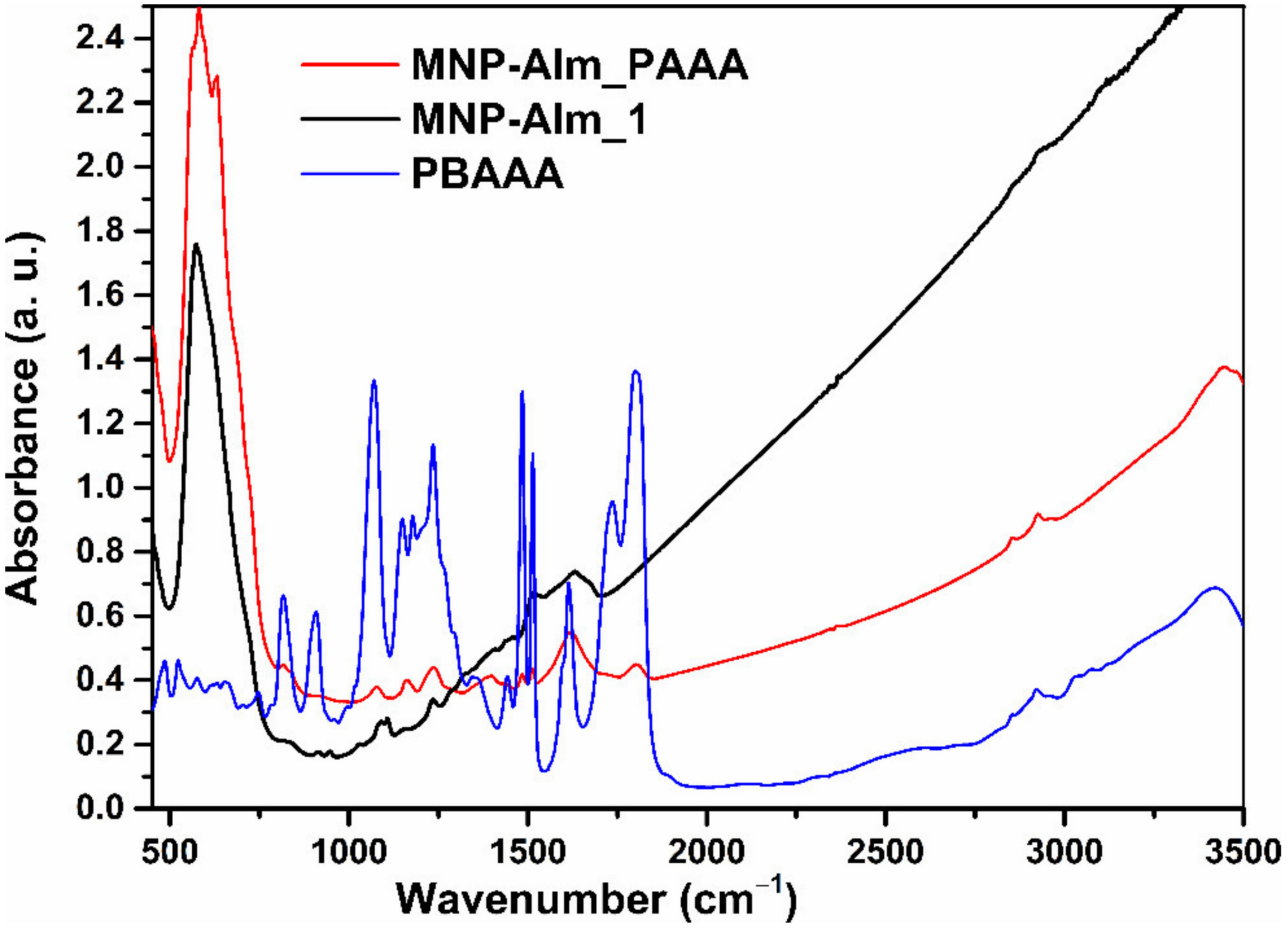
FTIR spectra of functionalized magnetic nanoparticles MNP-AIm_1, polymer PBAAA and magnetic nanostructure MNP-AIm_PAAA.

Figure 2 reveals important changes in the FTIR spectrum of amino-imidazole functionalized magnetic nanoparticles MNP-AIm_1 and the magnetic nanostructures of MNP-AIm_PAAA. The FTIR of MNP AIm_PAAA included specific absorption bands for both the magnetite and the polymer, demonstrating the attachment of the polymer to the MNP surface. The absorption band indicating the covalent bonding of the polymer on the MNP-AIm_1 surface was located at 1800 cm^−1^ for MNP-AIm_PAAA and was assigned to the C=O band from the benzofuranone ring. The intensity of the benzofuranone C=O band at 1800 cm^−1^ was dramatically reduced compared to the non-linked PBAAA, demonstrating the transformation of the lactone into amide moieties, i.e., a covalent bond. The amide bond has a broad absorption band at 1620 cm^−1^. The shoulder at 1730 cm^−1^ in the MNP-AIm_PAAA spectrum can be attributed to the carboxyl moiety. The two weak absorption bands at 2856 and 2921 cm^−1^ in the FTIR spectra of the polymer PBAAA and the magnetic nanostructure AIm_PAAA, respectively, were ascribed to the aliphatic CH-groups and the stretching-mode band for the =C-H bond. The split in the FTIR spectrum of MNP-AIm_PAAA at 585 cm^−1^ and 630 cm^−1^ wave numbers was due to the principal effect of the nanosized iron oxide particles, where a part of the polymer is interacting with the MNP’s surface. The unbinding of the many bonds from the surface atoms resulted in the rearrangement of localized electrons on the surface of the magnetite nanoparticles [23].

#### 3.2.2. X-ray Powder Diffraction

**Figure 3:**
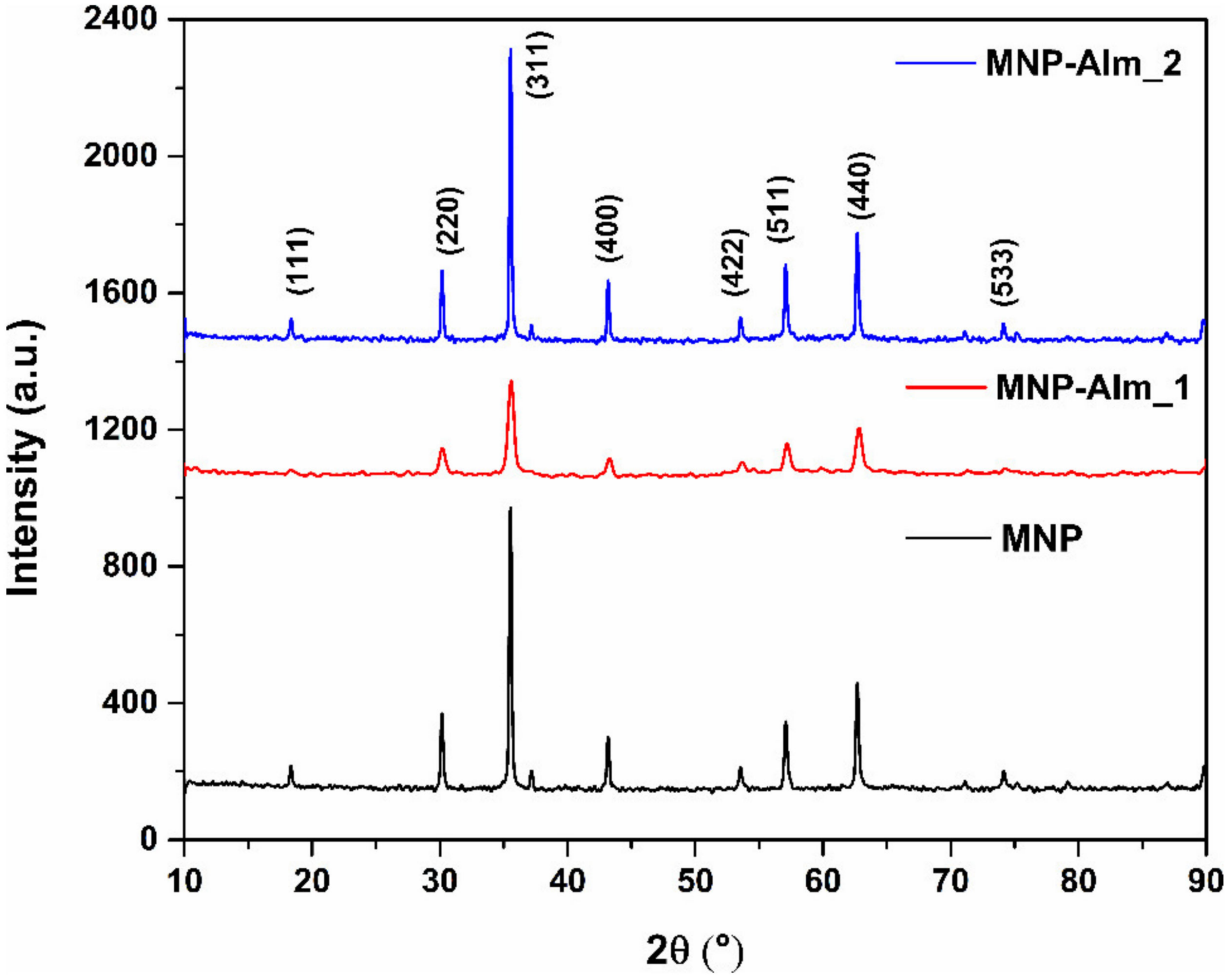
*X*-ray diffraction (XRD) patterns of magnetic nanoparticles MNP, MNP-AIm_1, and MNP-AIm_2.

The X-ray diffractograms of MNP, MNP-AIm_1, and MNP-AIm_2 were measured at room temperature and are presented in Figure 3. The diffraction peaks of (111), (220), (311), (400), (422), (511), (440), and (533) reflect the magnetite crystal with a cubic spinel structure. Considering all the results achieved in XRD using the Scherrer equation, a non-significant change was observed regarding the crystallite size. The uncovered MNP was obtained with a crystallite size of 34.5 nm, while the MNP-AIm_1 synthesized only in the presence of AIm, which had a crystallite size of 30 nm. A crystallite size of 34.2 nm was observed in MNP-AIm_2, which was synthesized in the presence of AIm and NaOH.

#### 3.2.3. Transmission Electron Microscopy

**Figure 4:**
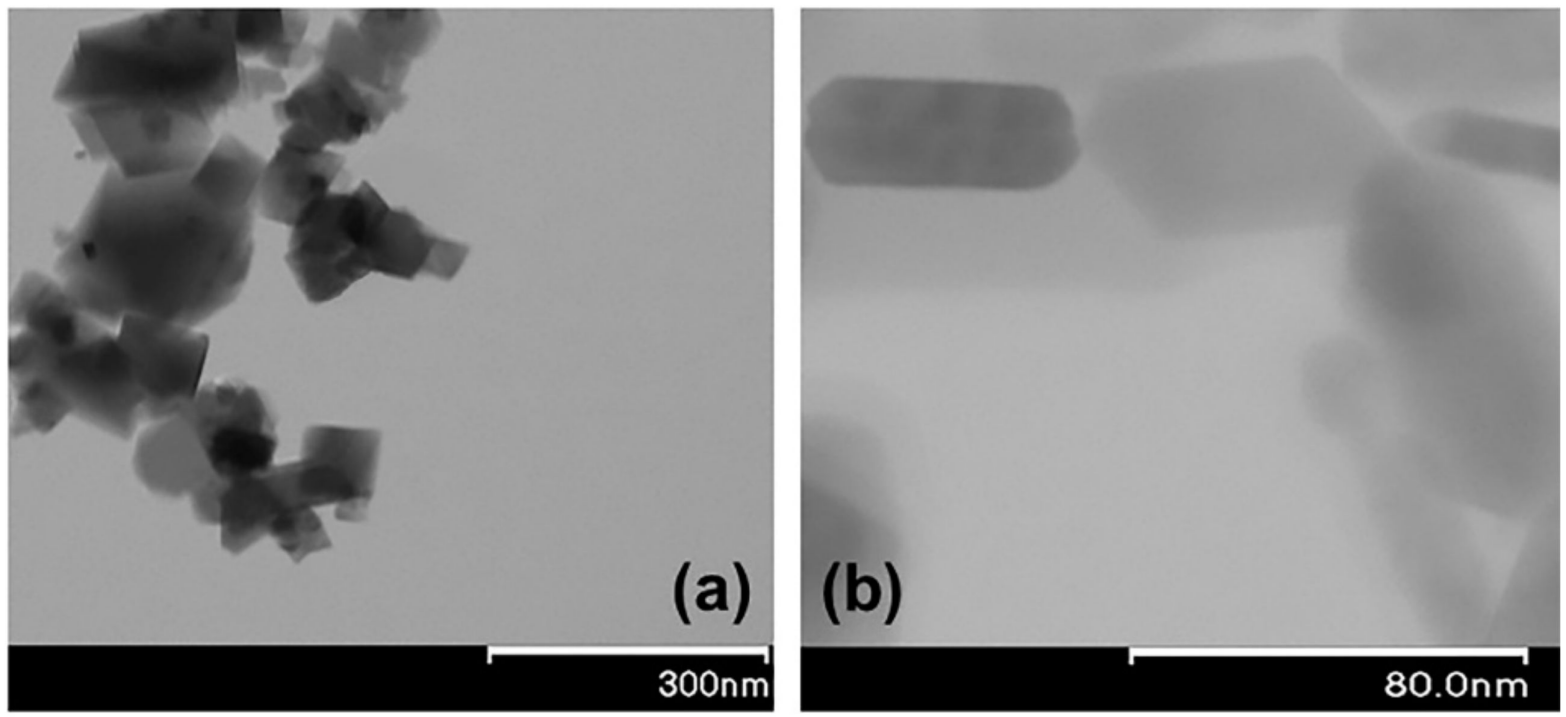
TEM image of uncovered MNP (**a**) and stabilized magnetic nanoparticles MNP-AIm_2 (**b**), obtained by depositing 10 µL of a suspension of each sample onto a copper grid coated with a thin carbon film and drying it.

**Figure 5:**
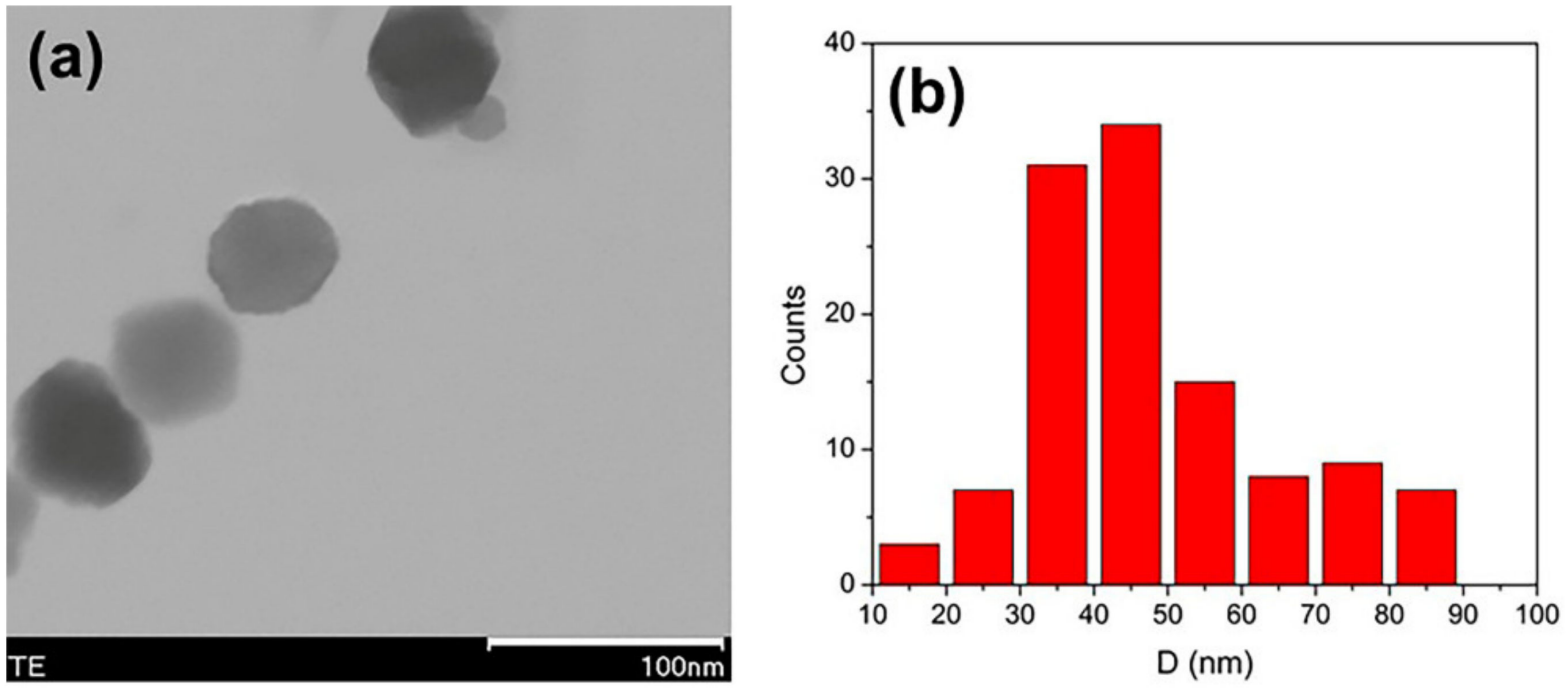
TEM image of stabilized magnetic nanoparticles MNP-AIm_1 (**a**) obtained by depositing 10 µL of a suspension of each sample onto a copper grid coated with a thin carbon film and drying it and size distribution (diameter) of the magnetic nanoparticles MNP-AIm_1 based on TEM measurements (**b**).

TEM investigations of MNP, MNP-AIm_1, and MNP-AIm_2 (Figure 4 and Figure 5) revealed different morphologies. The TEM images of MNP (Figure 4a) and MNP-AIm_2 (Figure 4b) form suspensions with partial aggregation, but in the case of MNP-AIm_1 (Figure 5a), TEM images reveal an excellent dispersion of the magnetite nanoparticles.

The synthesis conditions strongly influence the morphology of the magnetic nanoparticles: MNP contained spherical and cubic nanoparticles (Figure 4a), MNP-AIm_1 displayed spherical nanoparticles with a mean diameter D = 45 nm (Figure 5). In comparison, rod-like nanoparticles with a mean length of 70 nm characterized the sample MNP-AIm_2 (Figure 4b). It seems that the synthesis in the presence of NaOH, as used in the preparation of MNP-AIm_2, favored the appearance of the nanoparticles’ morphologies differently from the spherical ones found in MNP-AIm-1.

#### 3.2.4. X-ray Photoelectron Spectroscopy

**Figure 6:**
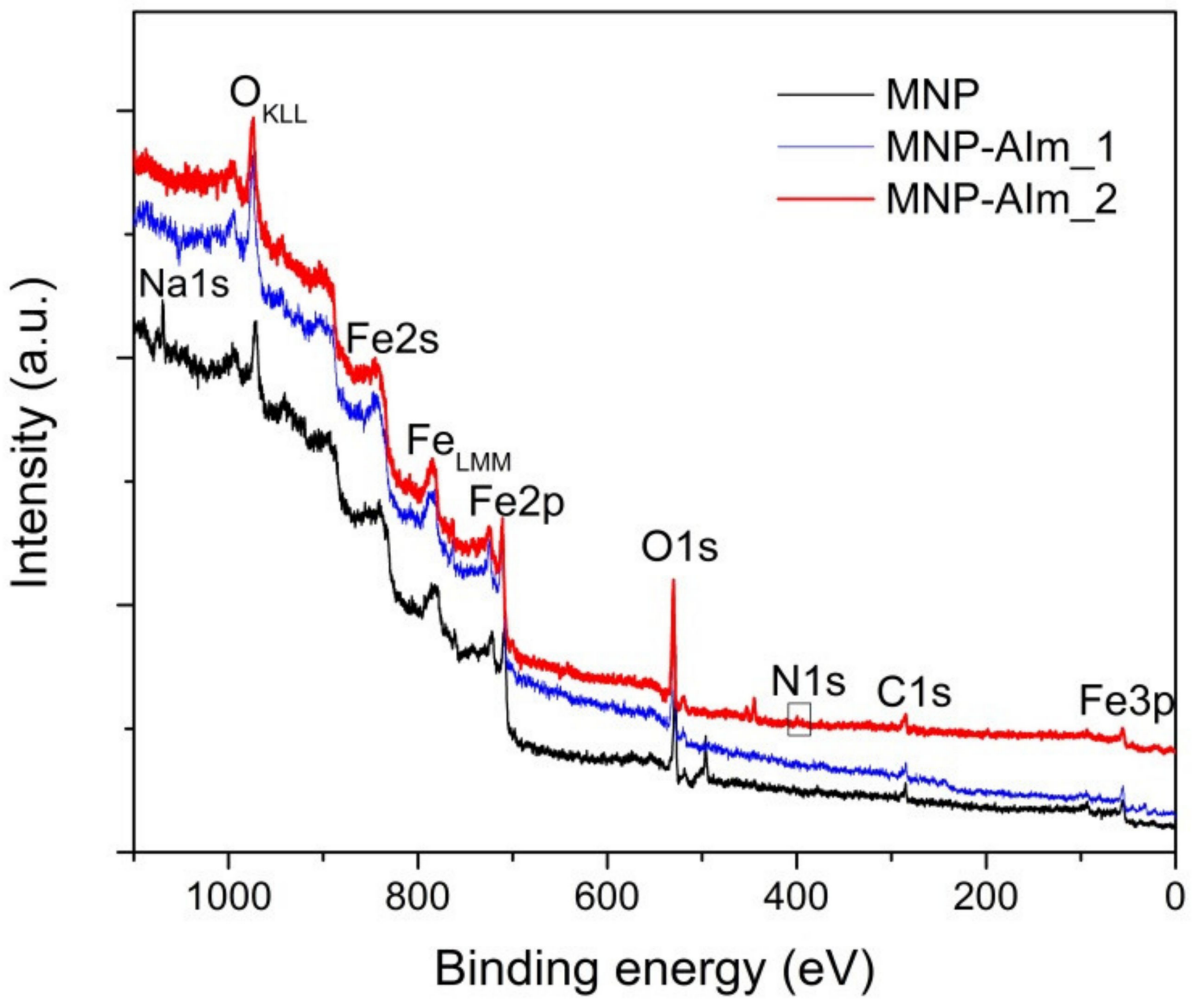
XPS survey spectra for MNP, MNP-AIm_1 and MNP-AIm_2.

The XPS survey spectra showed all element peaks of the MNP, MNP-AIm_1, and MNP-AIm_2 samples (Figure 6).

**Figure 7:**
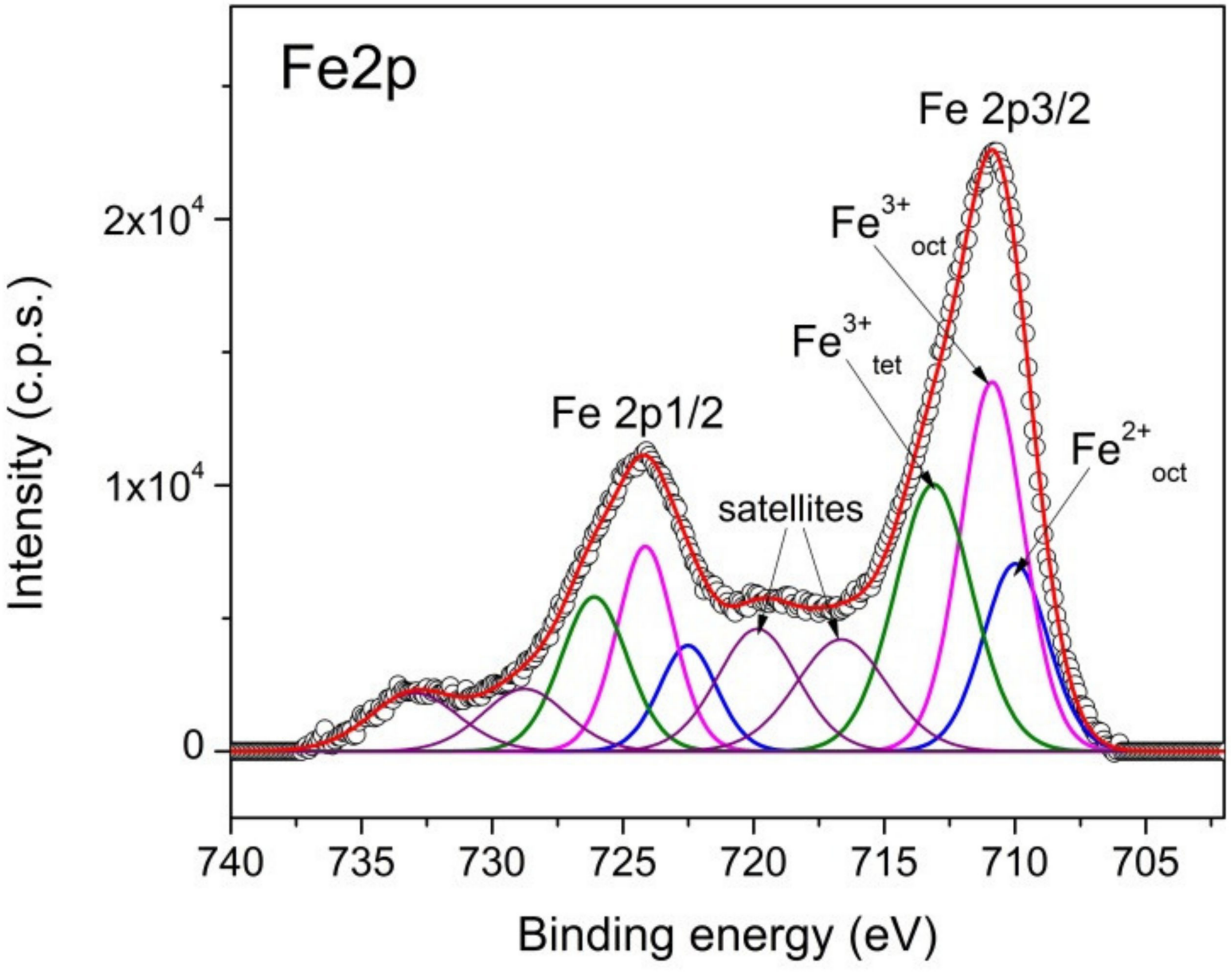
High-resolution XPS spectrum of Fe2p core levels for MNP.

The high-resolution XPS spectrum of Fe2p for MNP shown in Figure 7 included the doublet Fe2p3/2 and Fe2p1/2. The components corresponding to Fe^2+^ octahedral, Fe^3^+ octahedral, and Fe^3+^ tetrahedral, respectively, and the satellites provided the best fit for the Fe2p spectrum [24,25]. We determined the ratio of the atomic concentration Fe^3+^/Fe^2+^ = 2.3 from the Fe^3+^ and Fe^2+^ peak areas; this value is close to that expected for magnetite. Similar XPS spectra of Fe2p were obtained for the samples MNP-AIm_1, MNP-AIm_2, and MNP-AIm_PAAA.

**Figure 8:**
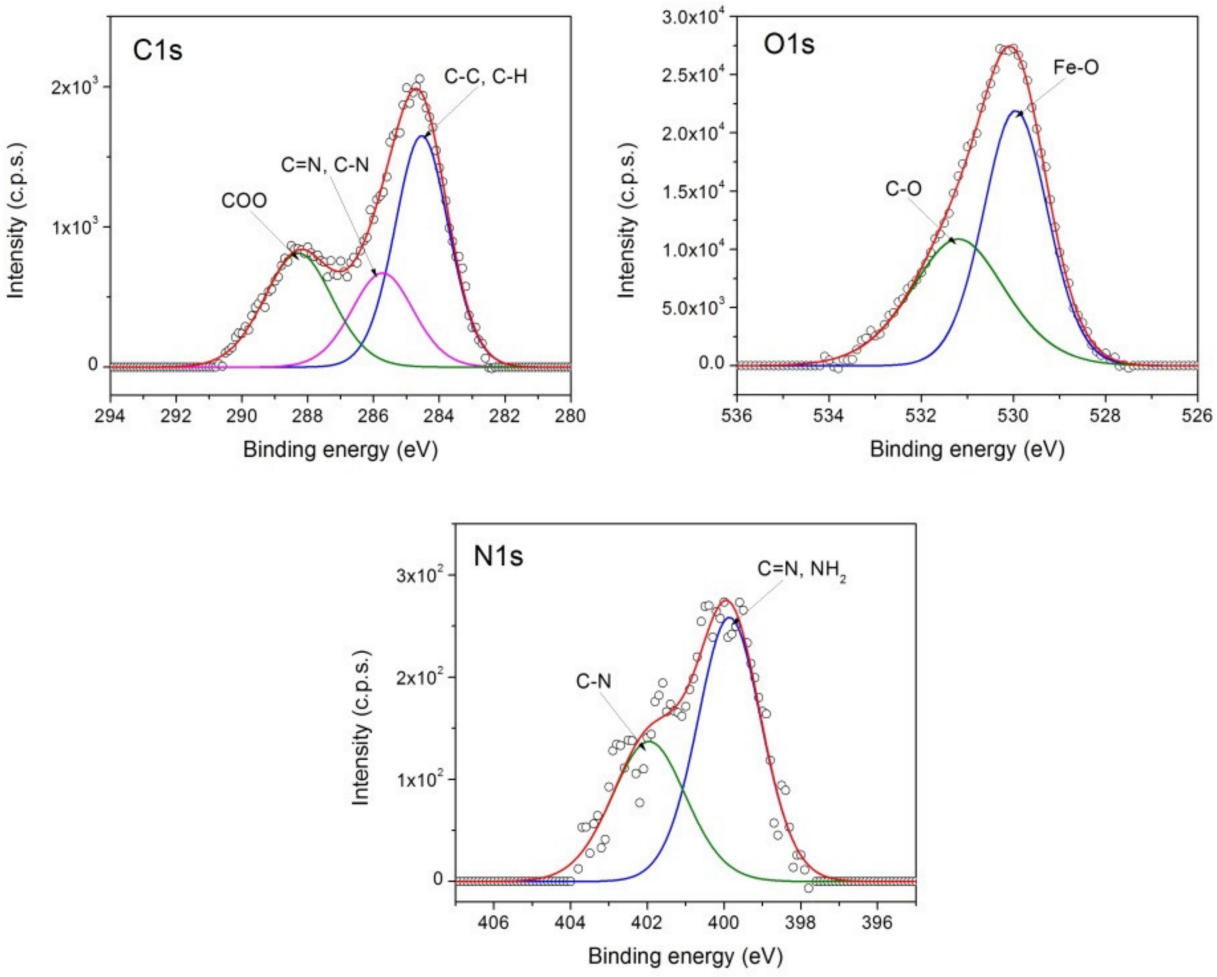
High-resolution XPS spectra of C1s, O1s and N1s core-levels for MNP-AIm_1.

**Figure 9:**
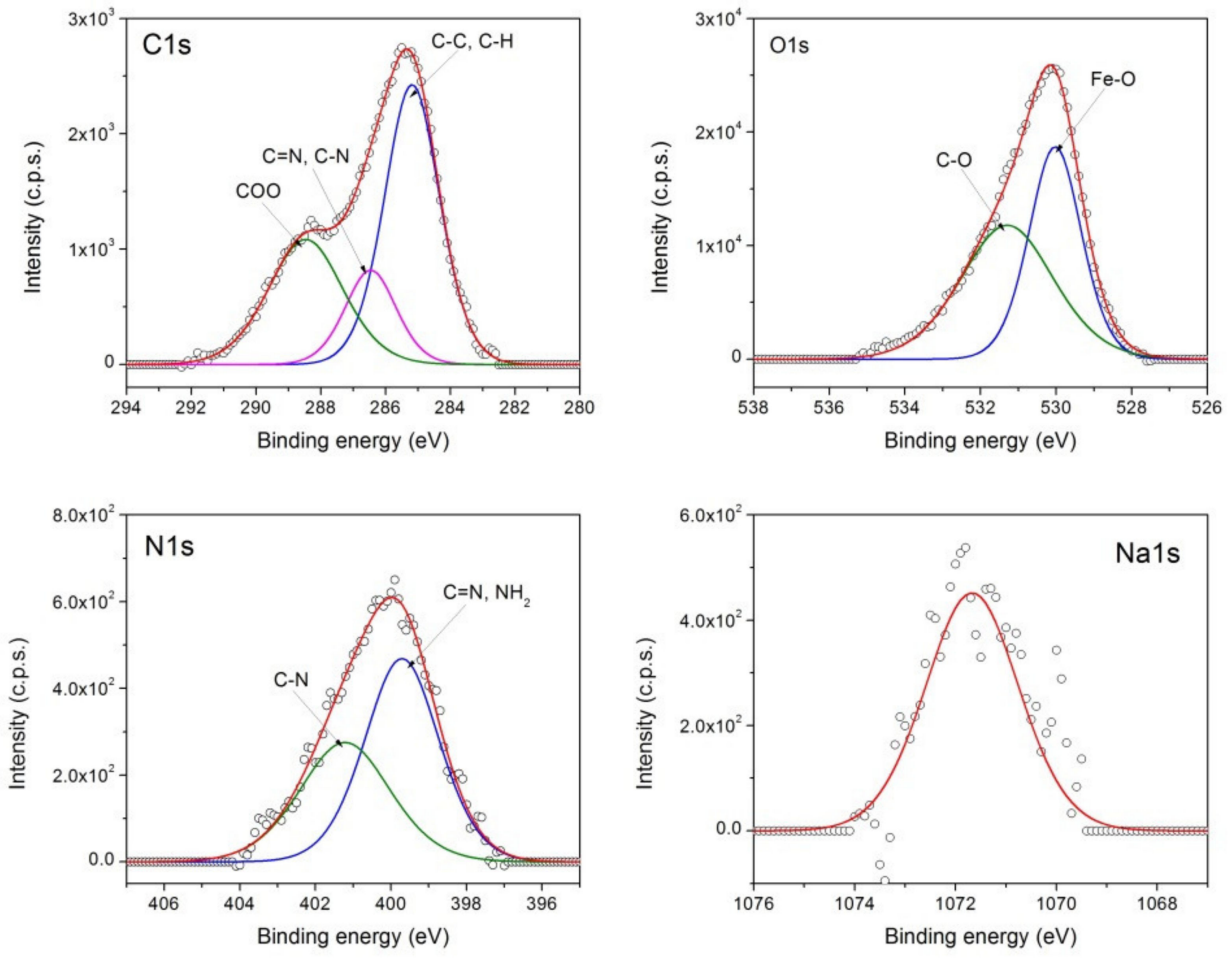
High-resolution XPS spectra of C1s, O1s, N1s and Na1s core-levels for MNP-AIm_2.

In Figure 8 and Figure 9, the high-resolution XPS spectra of the C1s, O1s, N1s, and Na1s core levels for samples MNP-AIm_1 and MNP-AIm_2, respectively, are presented. The deconvolution of the XPS peaks of the C1s, O1s, and N1s in Figure 8 and Figure 9 showed similar peaks. 

The best fit for the C1s spectrum was obtained with three components assigned to the carbon atoms from C-C, CH (284.5 eV), C=N, C-N (285.7 eV) groups characteristic to 1-(3-aminopropyl)imidazole, and COO (288.3 eV) due to environmental contamination. The O1s spectrum exhibited two components: one located at 530 eV ascribed to Fe-O from magnetite and the other located at 531 eV from C-O, which are also associated with contamination. The N1s spectrum showed two peaks assigned to C=N, NH_2_ (399.8 eV), and C-N (401.9 eV), which are characteristic of imidazole [26]. 

The XPS spectra from Figure 8 and Figure 9 demonstrate the coating of the magnetite nanoparticles with 1-(3-aminopropyl)imidazole. Moreover, the sample MNP-AIm_2 contained Na, which was adsorbed in the sample due to the reaction conditions. 

The atomic concentrations (%) calculated from the XPS spectra for MNP-AIm_1 and MNP-AIm_2 given in Table 1 highlights an increase in the C concentration for the sample MNP-AIm_2 as compared with MNP-AIm_1, indicating a higher degree of coating of the nanoparticles with 1-(3-aminopropyl)imidazole MNP-AIm_2.

**Figure 10:**
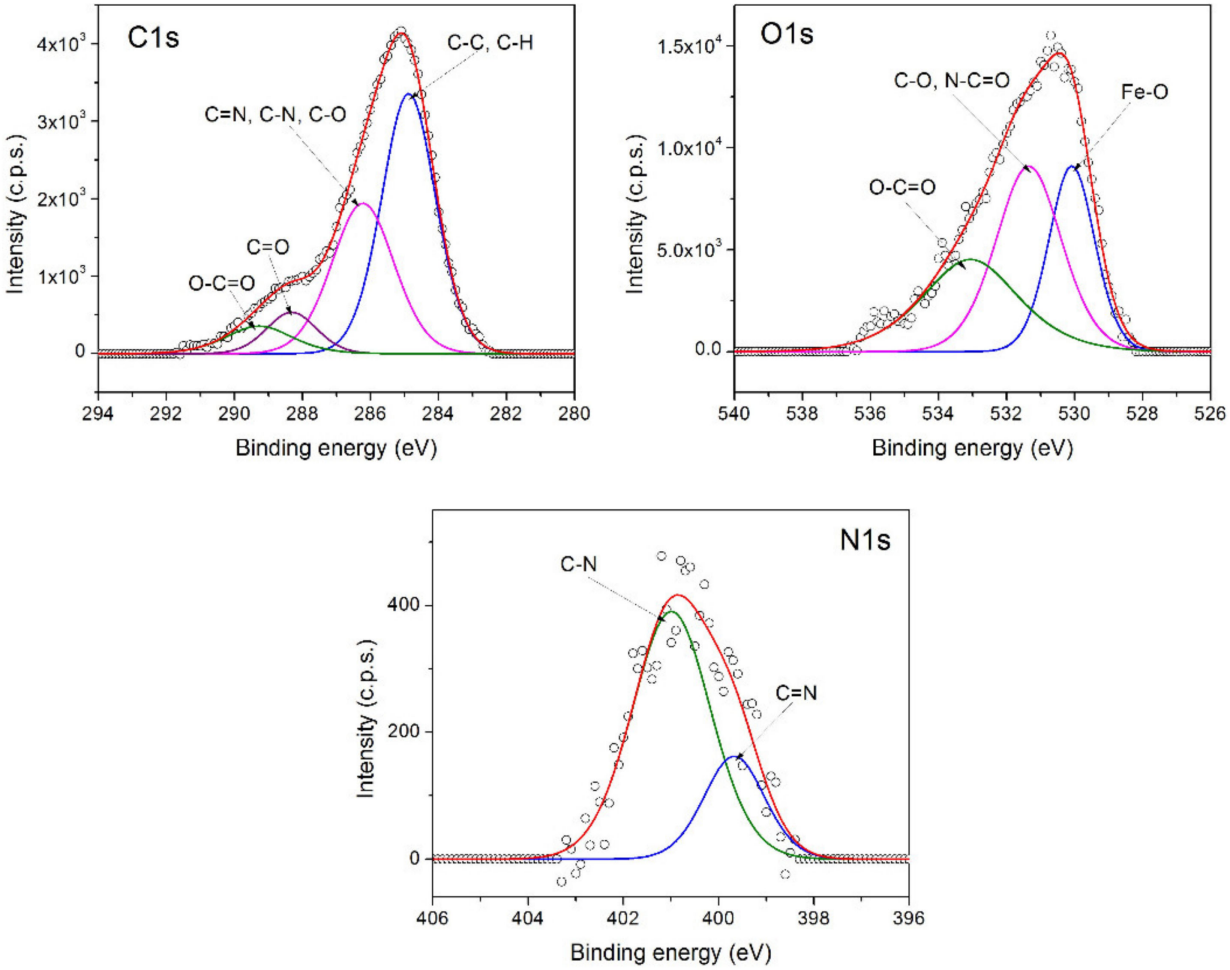
High-resolution XPS spectra of C1s, O1s and N1s core-levels of MNP-AIm_PAAA.

The high-resolution XPS spectra of the C1s, O1s, and N1s core levels for the sample MNP-AIm_PAAA are shown in Figure 10. The deconvolution of the C1s spectrum from Figure 10 shows four peaks characteristic of the polymer coating layer: C-C, C-H (284.9 eV); C=N, C-N, C-O (286.2 eV); C=O (288.32 eV); O-C=O 289.28 eV). The O1s spectrum contained three components: 530.09 eV (Fe-O); 531.35 eV (C-O, N-C=O); 533.07 eV (O-C=O). The best fit of the N1s spectrum from Figure 10 was achieved with two components ascribed to C=N (399.68 eV) and C-N (401 eV); the intensity ratio of these components was different from those from Figure 8 and Figure 9 due to the poly(benzofurane-*co*-arylacetic acid) coupling on the surface of the amino-functionalized magnetic nanoparticles. 

A comparison of the atomic concentrations (%) calculated from the XPS spectra (Table 1) shows a high C concentration increase for the sample MNP-AIm_PAAA, evidence of the polymer coating of functionalized magnetic nanoparticles.

#### 3.2.5. Magnetic Properties

**Figure 11:**
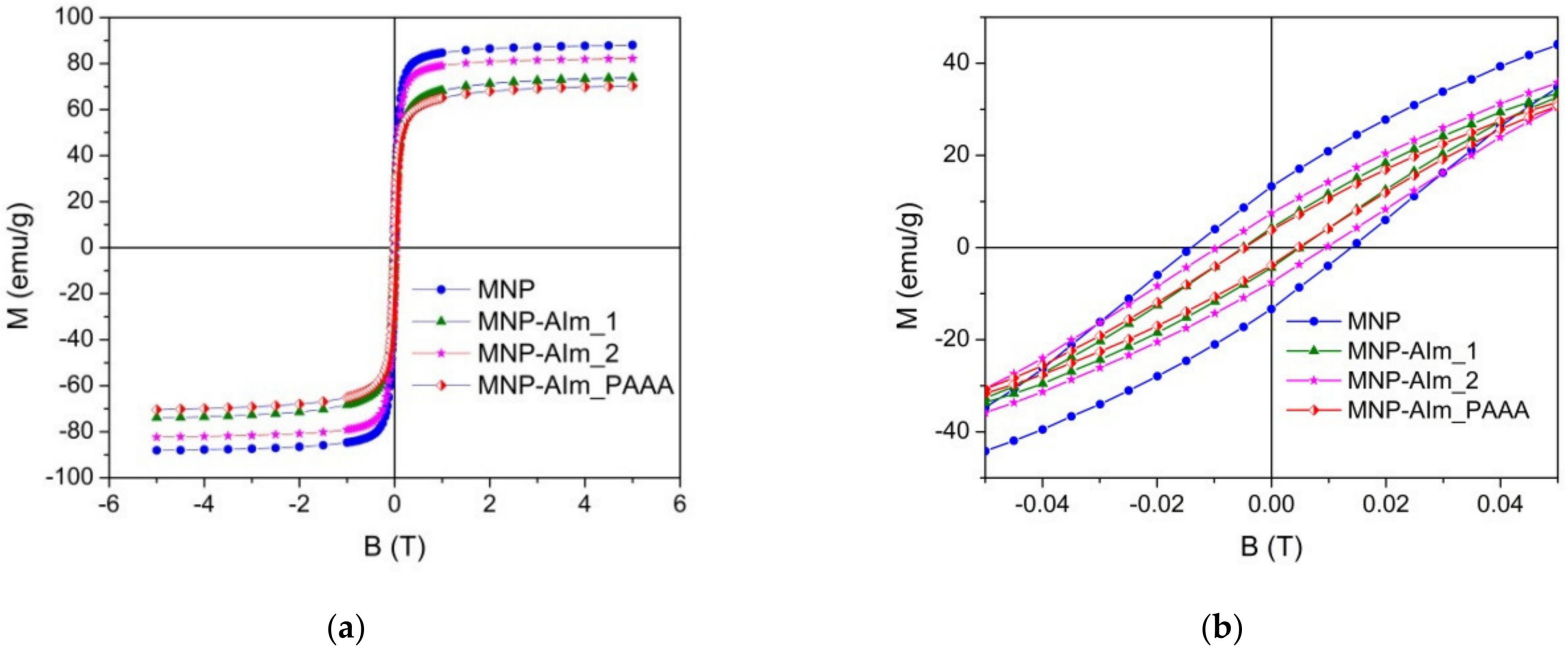
The magnetization (**a**) vs. applied magnetic field (**b**) of uncoated MNP and magnetic nanostructures MNP-AIm_1, MNP-AIm_2 and MNP-AIm_PAAA at room temperature.

The magnetization curves for MNP, for functionalized nanoparticles MNP-AIm_1, MNP-AIm_2 and polymer-coated nanoparticles MNP-AIm_PAAA are shown in Figure 11. These samples featured astonishingly high saturation magnetization values and low coercivity (Table 2).

The higher saturation magnetization value for MNP-AIm_2 (82.8 emu/g) as compared to the one for MNP-AIm_1 (75.4 emu/g) was due to the difference in crystallite sizes.

#### 3.2.6. Thermogravimetric Analyses of Magnetic Nanoparticles

**Figure 12:**
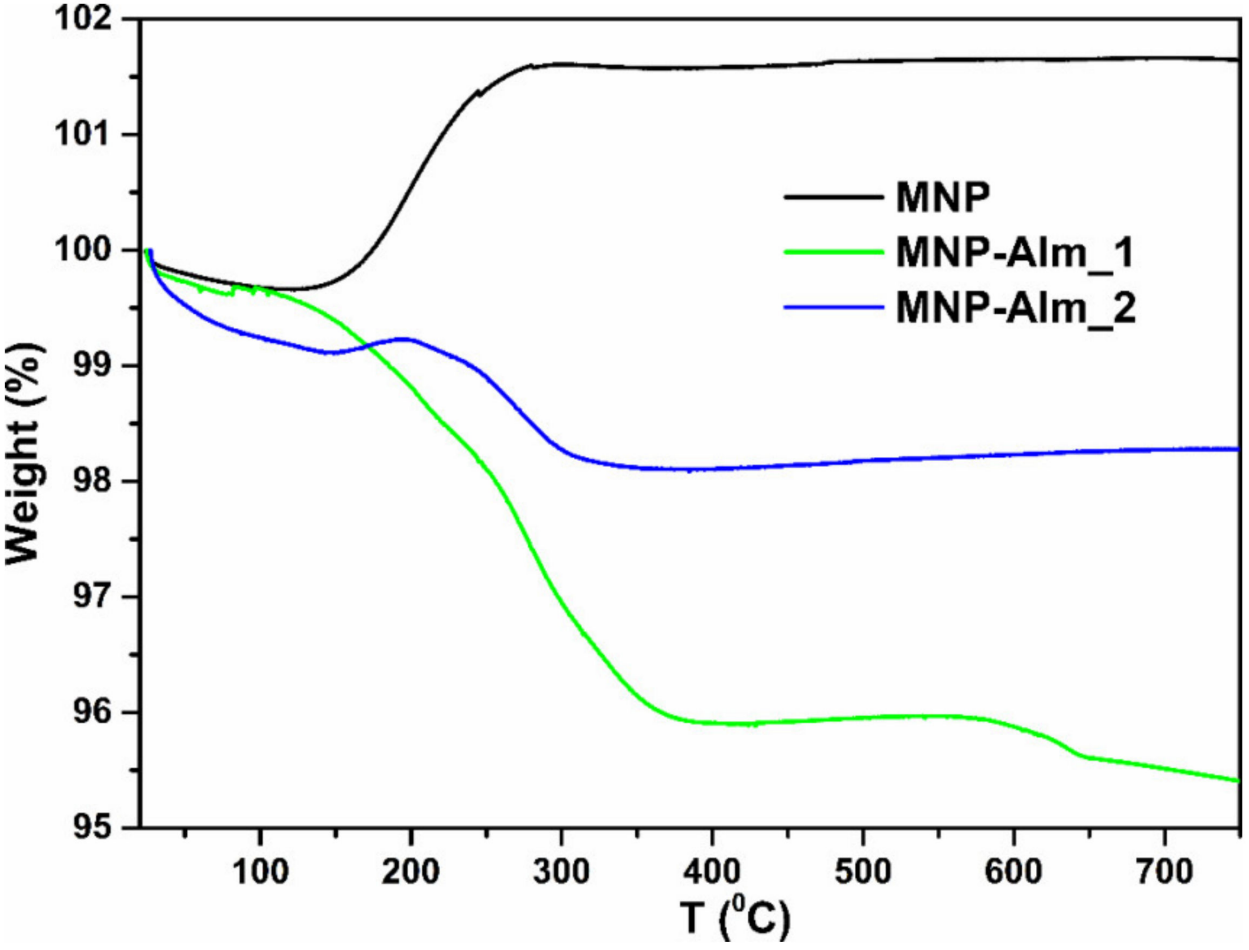
TGA curve of uncovered magnetic nanoparticles MNP, MNP-AIm_1, and MNP-AIm_2.

The Thermogravimetric Analyses (TGA) curves (Figure 12) demonstrate that MNP-AIm_2 contains more organic coating than MNP-AIm_1, thus confirming the conclusions drawn from the FTIR spectroscopy and XPS (see above). The mass increase seen in the uncoated MNP sample was due to the oxidation of magnetite (FeO · Fe_2_O_3_) to iron(III) oxide Fe_2_O_3_ due to the thermal degradation that occurred in the air’s atmosphere. Because the oxidation process is exothermic, the kinetic rate of the oxidation reaction of magnetite to hematite increased as the temperature rose. Therefore, the amount of oxygen produced by the Fe(II) to Fe(III) oxidation process increased proportionally with the amount of Fe(II) initially present [27,28].

## 4. Conclusions

A remarkably straightforward synthesis of functionalized MNP was found by treating aqueous solutions of FeCl_2_ with 1-(3-aminopropyl)imidazole in the air at room temperature. The structural characterization of the newly functionalized magnetic nanoparticles was carried out using FTIR and XPS spectroscopy. AIm ensures, on the one hand, an excellent fixation on the surface of MNPs through the imidazole ring, which is a powerful complexing agent, and, on the other side, offers functionalization with the amino groups of these nanoparticles, and all of this occurs in a short reaction time and at low cost. X-ray powder diffraction (XRD) and TEM data were used to examine the form and size of the as-prepared magnetite nanoparticles, and the drawn conclusion is that depending on the chosen base, we prepared nanoparticles with a defined, spherical, cubic or rod-like shape. The magnetization measurements recorded at room temperature were in good agreement with the data computed using the newly developed core–shell model, confirming the superparamagnetism of the synthesized MNPs. The high saturation magnetization values (Ms = 82.8 emu/g) of the as-prepared functionalized MNPs measured at ambient temperature, as well as their superparamagnetic behavior, make them attractive candidates for a variety of applications, including heat transfer, magnetic separation, and magnetic hyperthermia. Due to the presence of the free amine group on the MNP’s surface, they are excellent candidates for linking various applicatory functions. Exploiting this fact, MNP-AIm_1 and MNP-AIm_2 were used as a platform for PBAAA covalent linkage, increasing their stability, improving their colloidal stability. The ability of the amino groups on the MNP’s surface from MNP-AIm_1 to link different molecules enhances their potential candidacy for immobilization of various moieties with biological or catalytic importance.

## Figures and Tables

**Table 1:** The atomic concentrations (%) were calculated from XPS spectra for MNP-AIm_1, MNP-AIm_2 and MNP-AIm_PAAA.

Sample	Atomic Concentrations (%)				
	Fe	C	O	N	Na
MNP-AIm_1	46.95	11.891	40.326	0.833	-
MNP-AIm_2	41.253	16.213	40.437	1.841	0.256
MNP-AIm_PAAA	22.870	30.242	45.478	1.41	-

**Table 2:** The saturation magnetization (M_S_) and coercivity (B_c_) for the samples MNP, MNP-AIm_1, MNP-AIm_2 and MNP-AIm_PAAA.

Sample	MNP	MNP-AIm_1	MNP-AIm_2	MNP-AIm_PAAA
M_S_ (emu/g)	89	75.4	82.8	71.8
B_c_ (T)	0.01	0.005	0.01	0.005

## Data Availability

No new data were created or analyzed in this study. Data sharing is not applicable to this article.
